# Antimalarial stewardship programs are urgently needed for malaria elimination: a perspective

**DOI:** 10.1051/parasite/2019016

**Published:** 2019-03-22

**Authors:** Anne-Lise Bienvenu, Abdoulaye Djimdé, Stéphane Picot

**Affiliations:** 1 Groupement Hospitalier Nord, Service Pharmacie, Hospices Civils de Lyon Lyon F-69004 France; 2 ICBMS CNRS 5246, SMITh, Malaria Research Unit, Campus Lyon-Tech La Doua, Université de Lyon Villeurbanne F-69100 France; 3 Malaria Research and Training Center, University of Sciences, Techniques and Technologies of Bamako PO Box: 1805 Point G Bamako Mali; 4 Groupement Hospitalier Nord, Institut de Parasitologie et Mycologie Médicale, Hospices Civils de Lyon Lyon F-69004 France

**Keywords:** Malaria, Treatment, Stewardship, Antimalarial drug, Elimination, Guidelines

## Abstract

Global malaria cases have not been significantly reduced over the last three years although more than USD 3 billion was invested in malaria control and elimination. The reasons for this stagnation are highly complex and multi-factorial. It remains that almost three billion treatment courses were supplied over the period 2010–2017: 30% of them without malaria tests, and some with suboptimal doses leading to the risk of selection of resistant parasites. An antimalarial stewardship program should be implemented at the healthcare provider, physician, pharmacist, medical student, and population levels. This would significantly reinforce the impact of international guidelines and national malaria program policies and fill the gap between recommendations and actual practices.

## Abstract (fr)

Le nombre total de cas de paludisme n’a pas été significativement réduit au cours des trois dernières années bien que plus de 3 milliards de dollars aient été investis dans le contrôle et l’élimination du paludisme. Les raisons de cette stagnation sont complexes et multifactorielles. Néanmoins, presque trois milliards de traitement ont été fournis entre 2010 et 2017 : 30 % d’entre eux sans test de diagnostic du paludisme, certains avec des doses sub-optimales, entrainant le risque de sélection de parasites résistants. Un programme de gouvernance ou « stewardship » devrait être mis en place au niveau des prestataires de santé, des médecins, des pharmaciens, des étudiants en médecine et de la population. Cela renforcerait significativement l’impact des directives internationales et des politiques des programmes nationaux de lutte contre le paludisme, et comblerait le fossé entre les recommandations et les pratiques réelles.

Drug stewardship is an activity that includes appropriate selection, dosing, route, and duration of therapy. Its primary goal is to optimize clinical outcomes while minimizing unintended consequences of drug use, including toxicity, selection of pathogenic organisms, and the emergence of resistance. Antimicrobial stewardship programs have been widely implemented with significant impact in the fight against infectious diseases caused by multiple organisms, including bacteria [6] and fungi [1]. Parasites are also responsible for lethal diseases and have a significant impact on the exposed populations. Among parasitic infections, malaria caused 435,000 deaths in 2017, making it the most deadly [8]. The fight against malaria is facing many challenges, including the continued emergence of parasite resistance to antimalarial drugs [5]. Monitoring the efficacy of drugs has been a continuous activity for researchers, National Malaria Programs, and international organizations such as the World Health Organization (WHO) and more specifically the World Wide Antimalarial Resistance Network (WWARN). Malaria was therefore included in the WHO 2030 Agenda for Sustainable Development. This declaration called for strategies to begin to reverse the global incidence of malaria. A novel approach to combat malaria in a world where nearly half of the population is at risk of malaria is antimalarial stewardship. Antimalarial stewardship programs (AMSPs) should be hospital-based, implemented at the local level, and adapted to local conditions with the objective of increasing the frequency of correct prescribing of antimalarial drugs. It requires local leadership commitment and high flexibility in implementation [2].

Why are AMSPs needed in malaria-endemic areas?

1. Because progress in global malaria control has stalled.

According to WHO’s latest World malaria report, an estimated 219 million cases of malaria occurred worldwide in 2017, compared with 217 million cases in 2016; the estimated number of deaths from malaria was 435,000, compared to 451,000 in 2016 [8]. During this period, mortality rates stalled in South-East Asia, the Western Pacific and Africa, and increased in the Eastern Mediterranean and the Americas [8].

1. Because management is not equal for all cases

According to WHO, malaria case management consists of prompt diagnosis and effective treatment, a vital component of malaria control and elimination strategies [7]. However, antimalarial treatments are still often initiated without confirmation of diagnosis, leading to unnecessary antimalarial selective pressure. Rapid diagnostic tests are implemented in most healthcare centers, but are not always used for treatment decision-making. Moreover, although protocols for effective treatment do exist they are not always updated in all malaria-endemic countries, and recommended drugs may not be available. The use of combination therapy and weight-based dosing, ensuring optimal exposure of patients to drugs, are of particular importance to ensure treatment effectiveness.

1. Because incorrect dosing is frequent

Treatment compliance is difficult, particularly for patients who do not have easy access to healthcare facilities or who are not aware of the consequences of this. Furthermore, weight-based dosing is not always carried out and may be a contributor to suboptimal dosing. Counterfeit and substandard antimalarial drugs, that contain no active ingredient, less than the required amount, or ingredients not described on the package, are also responsible for under- or over-dosing.

1. Because toxicity may occur

Over-dosing may lead to antimalarial toxicity in patients, but some toxicities are related to patient genotypes; for example, primaquine causes predictable oxidant hemolysis in those with glucose 6-phosphate dehydrogenase deficiency, for whom a specific drug regimen is required [7].

1. Because antimalarial resistance is increasing

Resistance poses one of the greatest threats to malaria control and is responsible for increased malaria-related morbidity and mortality. Under-dosing of antimalarial drugs contributes in part to an increase in resistance. This threat is particularly alarming as the use of artemisinin-based combination therapy, the gold standard antimalarial, was demonstrated to be associated with slow parasitic clearance in South-East Asia [4].

1. Because we are on the way towards malaria elimination

The main objective of AMSPs should be resistance containment, which is essential for malaria eradication. AMSPs will therefore contribute to the elimination process.

Implementing AMSPs in endemic countries is ambitious, but not overwhelming. Antimalarial treatments should be considered differently at the international, national and local levels. Experts from international organizations are highly committed to the preparation and dissemination of guidelines defining the best way to treat patients with malaria. These guidelines are taken into account at the national level, mostly by the National Malaria Programs or equivalents under the supervision of the Ministry of Health. These national bodies define the first and second line treatments for the country, according to international recommendations, and national consideration in terms of market or procurement.

The local level is diverse since malaria treatments are given from the public sector, the private sector, and not-for-profit providers. Physicians and pharmacists may face other challenges such as interruption in the supply chain, fake drugs, and drugs not following national health policies. These problems may be responsible for periodic drifts in malaria treatment, leading to a high risk of misuse of drugs for various periods of time in limited and disperse areas. These unpredictable events may be under the radar of national and international bodies, leading to uncontrolled drug exposure of parasites. This is the main reason why AMSPs are urgently needed. AMSPs are the only tool to limit the frequent drifts or misuses of antimalarial drugs.

As recommended by the US Centers for Disease Control and Prevention [1], leadership commitment is a core element and needs to be achieved in each level; AMSP leaders should be responsible for dissemination of key messages targeting physicians and pharmacists, and training of medicine and pharmacy students in line with identified policy challenges. Strengthening the link between physicians and pharmacists with the goal of optimizing antimalarial treatment is also expected to be a substantial part of AMSPs. Moreover, international collaborations will be beneficial to AMSP implementation, as should be the involvement of public health authorities [3]. AMSPs are expected to be well perceived at the population level: populations are aware of the disease’s severity and there is a collective interest in malaria eradication. Education of the population through stewardship leaders would be a vital part of the success of AMSPs.

Local AMSPs will succeed and have a significant impact if there is a close relationship between AMCPs and Ministries of Health. These bodies will receive information from AMSP leaders about the on-going situation at the local level, and they can act at the national level to address the issues detected by field workers specifically involved in stewardship programs. This link is essential for the sustainability of AMSPs.

More generally, we could also expect that AMSP implementation will bring new expertise to healthcare personnel involved in the field of parasitic diseases, which will contribute positively to efforts made to control other parasitic diseases, including neglected tropical diseases such as Chagas disease, schistosomiasis, and soil-transmitted parasitic diseases.
